# Supplementary material for: ProteinShader: illustrative rendering of macromolecules
Source: BMC Struct Biol. 2009 Mar 30;9:19. doi: 10.1186/1472-6807-9-19 (PMC2672931; doi:10.1186/1472-6807-9-19)
Supplement: Additional file 1 — ProteinShader program without source code. This compressed file contains the complete ProteinShader program including associated libraries, but no source code. A README.txt file gives an overview of the ProteinShader distribution, and the index.html file in the help subdirectory has directions on getting started with the program as well as a set of tutorials. [file 1472-6807-9-19-S1.zip › ProteinShader-beta-0_9_4-binary/help/api/org/proteinshader/graphics/typography/Glyph.html]

Glyph (ProteinShader API)


|  |  |  |
| --- | --- | --- |
| **Overview** \| **Package** \| **Class** \| **Use** \| **Tree** \| **Deprecated** \| **Index** \| **Help** | |  |
| **PREV CLASS**   **NEXT CLASS** | **FRAMES**    **NO FRAMES**     **All Classes** |  |
| SUMMARY: NESTED \| FIELD \| CONSTR \| METHOD | DETAIL: FIELD \| CONSTR \| METHOD |  |


---


## org.proteinshader.graphics.typography Class Glyph

```
java.lang.Object
  org.proteinshader.graphics.typography.Glyph
```

---

``` public class Glyph extends Object ```

Stores a 2D array of bytes that form a graphical representation of a
character. The bytes are ultimately intended to be copied into a
region of an OpenGL texture object.

---

**Constructor Summary**

`Glyph(String name,
int x1,
int y1,
int x2,
int y2,
int bytesPerPixel)`
  
          Constructs a Glyph.


| **Method Summary** | |
| --- | --- |
| `void` | `extractGlyphImage(byte[][] glyphSetImage)`             Extracts the glyph from the texture image given as an argument. |
| `int` | `getBytesPerPixel()`             Returns the number of bytes per pixel, which should be in the range of 1 to 4. |
| `int` | `getHeight()`             Returns the height of the glyph in pixels. |
| `byte[][]` | `getImage()`             Returns the 2D byte array with the glyph image in it. |
| `String` | `getName()`             Returns the name of the glyph. |
| `int` | `getWidth()`             Returns the width of the glyph in pixels. |
| `int` | `getX1()`             Returns the x-coordinate of the top left corner of the glyph (this coordinate is used for extracting the glyph from the larger image file with several glyphs). |
| `int` | `getX2()`             Returns the x-coordinate of the bottom right corner of the glyph (this coordinate is used for extracting the glyph from the larger image file with several glyphs). |
| `int` | `getY1()`             Returns the y-coordinate of the top left corner of the glyph (this coordinate is used for extracting the glyph from the larger image file with several glyphs). |
| `int` | `getY2()`             Returns the y-coordinate of the bottom right corner of the glyph (this coordinate is used for extracting the glyph from the larger image file with several glyphs). |

**Methods inherited from class java.lang.Object**

`clone, equals, finalize, getClass, hashCode, notify, notifyAll, toString, wait, wait, wait`

**Constructor Detail**

### Glyph

```
public Glyph(String name,
             int x1,
             int y1,
             int x2,
             int y2,
             int bytesPerPixel)
```

:   Constructs a Glyph.


**Method Detail**

### extractGlyphImage

```
public void extractGlyphImage(byte[][] glyphSetImage)
                       throws GlyphImageException
```

:   Extracts the glyph from the texture image given as an argument.
      
      
    The glyphSetImage argument is a 2D array of bytes that will
    normally hold a large set of glyphs. The rectangular region of
    bytes that is extracted will be determined by the (x1, y1) and
    (x2, y2) coordinates given the constructor along with the number
    of bytes per pixel.

    :   **Parameters:**: `glyphSetImage` - an image containing a set of glyphs. **Throws:**: `GlyphImageException` - if the glyph image cannot be extracted from the larger glyph set image.

---


### getImage

```
public byte[][] getImage()
```

:   Returns the 2D byte array with the glyph image in it.
      
      
    The extractGlyphImage() method must have been called before this
    method is called (or the 2D array returned will be null).

    :   **Returns:**: The 2D byte array.

---


### getName

```
public String getName()
```

:   Returns the name of the glyph. The name is usually only one
    character, but a glyph can sometimes represent two or more
    characters.

    :   **Returns:**: The name as a String.

---


### getX1

```
public int getX1()
```

:   Returns the x-coordinate of the top left corner of the glyph
    (this coordinate is used for extracting the glyph from the
    larger image file with several glyphs).

    :   **Returns:**: The x-coordinate of the top left corner of the glyph.

---


### getY1

```
public int getY1()
```

:   Returns the y-coordinate of the top left corner of the glyph
    (this coordinate is used for extracting the glyph from the
    larger image file with several glyphs).

    :   **Returns:**: The y-coordinate of the top left corner of the glyph.

---


### getX2

```
public int getX2()
```

:   Returns the x-coordinate of the bottom right corner of the glyph
    (this coordinate is used for extracting the glyph from the
    larger image file with several glyphs).

    :   **Returns:**: The x-coordinate of the bottom right corner of the glyph.

---


### getY2

```
public int getY2()
```

:   Returns the y-coordinate of the bottom right corner of the glyph
    (this coordinate is used for extracting the glyph from the
    larger image file with several glyphs).

    :   **Returns:**: The y-coordinate of the bottom right corner of the glyph.

---


### getBytesPerPixel

```
public int getBytesPerPixel()
```

:   Returns the number of bytes per pixel, which should be in the
    range of 1 to 4.

    :   **Returns:**: The number of bytes per pixel.

---


### getWidth

```
public int getWidth()
```

:   Returns the width of the glyph in pixels.

    :   **Returns:**: The width in pixels.

---


### getHeight

```
public int getHeight()
```

:   Returns the height of the glyph in pixels.

    :   **Returns:**: The height in pixels.


---


|  |  |  |
| --- | --- | --- |
| **Overview** \| **Package** \| **Class** \| **Use** \| **Tree** \| **Deprecated** \| **Index** \| **Help** | |  |
| **PREV CLASS**   **NEXT CLASS** | **FRAMES**    **NO FRAMES**     **All Classes** |  |
| SUMMARY: NESTED \| FIELD \| CONSTR \| METHOD | DETAIL: FIELD \| CONSTR \| METHOD |  |


---

# *Copyright © 2007-2008*
